# Increased Weekly Mean PM_2.5_, and NO_2_ Are Associated With Increased Proportions of Lower Airway Granulocytes in Ontario Horses

**DOI:** 10.3389/fvets.2020.00185

**Published:** 2020-05-05

**Authors:** Gabrielle Brankston, Amy L. Greer, Quinn Marshall, Brittany Lang, Kai Moore, Douglas Hodgins, John T. G. Hennessey, Janet Beeler-Marfisi

**Affiliations:** ^1^Department of Population Medicine, Ontario Veterinary College, University of Guelph, Guelph, ON, Canada; ^2^Department of Pathobiology, Ontario Veterinary College, University of Guelph, Guelph, ON, Canada; ^3^Hennessey Equine Veterinary Professional Corp., Rockwood, ON, Canada

**Keywords:** ambient air pollution, smog, respirable particulate, air quality health index, case-crossover analysis, bronchoalveolar lavage, inflammatory airway disease, equine asthma

## Abstract

Ambient pollution is associated with the development and exacerbation of human asthma, but whether air pollution exposure is associated with lower airway inflammation in horses has not been fully evaluated. The Air Quality Health Index (AQHI) is an online tool used by asthmatic Ontarians to modify their outdoor activity when ambient pollution is high. A single AQHI value, falling on a scale from 1 to 10^+^, is calculated from measurements of fine particulate matter (PM_2.5_), nitrogen dioxide (NO_2_), and ozone (O_3_). Increased AQHI values predict an increased risk for presenting to a health care provider for assessment of asthma exacerbation, with a time lag of 0–9 days after an increase. Whether ambient air pollution is a risk factor for identifying increased lower airway inflammatory cells on cytologic evaluation of bronchoalveolar lavage fluid (BALF) of horses has not yet been explored. To investigate this relationship, case data including BALF cytology preparations from horses across southern Ontario, Canada, were retrieved from the Guelph Animal Health Laboratory's archives. Spanning the years 2007–2017, 154 cases were identified within a 41- by 30-km area surrounding the cities of Guelph and Kitchener. In 78 of 154 cases, cytologic reevaluation identified increased proportions of one or a combination of BALF neutrophils (mean 5%, range 0–15%), eosinophils (mean 2%, range 0–31%), and mast cells (mean 4%, range 0–10%). To assess the effect of lagged pollutant and temperature exposures in these 78 cases, weekly mean values of AQHI, PM_2.5_, NO_2_, O_3_, and temperature were recorded for the 4 weeks prior to the date of the horse's presentation for respiratory tract evaluation. The relationship between ambient exposures and increased proportions of lower airway granulocytes was evaluated using a case-crossover design. Single unit increases in 2-, and 3-week lagged weekly mean PM_2.5_ and NO_2_, were associated, respectively, with an 11% (*p* = 0.04, 95% confidence interval, CI = 1.01–1.22), and 24% (*p* = 0.03, 95% CI = 1.08–1.43) greater risk of identifying increased lower airway granulocytes. These findings suggest that exposure to increased ambient pollutants is associated with lower airway inflammation in Guelph and Kitchener area horses.

## Introduction

Inflammatory airway disease (IAD) is a common, performance-limiting, non-infectious disorder of the lower airways that can affect horses of any age, but is most commonly identified in younger sport horses (1–5). IAD shares similarities with allergic and non-allergic human asthma (6) including airway hyperresponsiveness, intermittent cough, increased lower airway inflammatory cells, and differing responses to therapy based on the predominant inflammatory cell type (3, 6, 7). Horses with IAD have increased proportions of lower airway granulocytes, including any one or a combination of neutrophils >5% but <25%, eosinophils >1%, and mast cells >2% (3). But, unlike neutrophils, there is no defined upper limit of eosinophils or mast cells (8–11) that would preclude a diagnosis of IAD. Subtypes of human asthma are characterized by the predominant lower airway inflammatory cell type identified in induced sputum samples (12). The subtypes that appear most similar to IAD are allergic asthma (>3% eosinophils), and the non-allergic forms of asthma including mixed granulocytic (>3% eosinophils) and paucigranulocytic [<3% eosinophils; (6, 12)]. Mast cells may play a role in the pathogenesis of some types of human asthma (13) but are not included in the diagnosis of the disease (7). Hemosiderophages have been noted in association with lower airway inflammation in horses (14–16), however, the relationship between distal airway inflammation and bleeding into the alveoli is incompletely understood (17). Severe forms of human asthma can be associated with pulmonary blood vessel inflammation (18), but there is no record of an association between blood vessel inflammation and alveolar hemorrhage in humans. Finally, horses of any age with IAD spontaneously recover (3), whereas, complete remission of human asthma is observed only in a subset of pubescent children (19); the disease is otherwise chronic with variably severe clinical signs over time (7).

The role of ambient pollution in the pathogenesis of IAD has not been thoroughly investigated (20–22). In humans, exposure to increased concentrations of ambient pollutants increases the risk of developing (23, 24) or exacerbating asthma (25, 26). In Ontario, the major ambient pollutants identified in association with asthma exacerbation include fine particulate matter of a median aerodynamic diameter ≤ 2.5 microns (PM_2.5_), nitrogen dioxide (NO_2_), and ozone [O_3_; (27)]. Nitrogen dioxide and PM_2.5_ mainly originate from combustion sources, but O_3_ is sunlight-dependent as it is created by a photochemical reaction involving nitrogen oxides (28). The World Health Organization guidelines for air pollution (WHO) indicate that PM_2.5_ and O_3_ likely have independent effects on health at pollutant concentrations found in developed nations (28), and a recent meta-analysis also suggested an independent effect on health by NO_2_ (29).

The effects of NO_2_ on lung health have not been examined in horses, but stabled Thoroughbreds exposed to higher concentrations of PM_2.5_ had increased tracheal mucus as a marker of lower airway inflammation (20). In children, exposure to NO_2_ (23, 24) and PM_2.5_ (24) have been associated with the development of asthma. Asthmatic children were more sensitive than adults to the acute effects of PM_2.5_, particularly during warmer weather (30). Increased susceptibility to the deleterious effects of exposure to diverse pollutants is common among children (23, 24, 30). This predisposition may relate to engaging in more vigorous outdoor activity, mouth breathing, and a lower nasal filtration capacity compared to adults (24). Experimental exposure of horses to O_3_ resulted in increased free iron in bronchoalveolar lavage fluid (BALF) and evidence of lower airway inflammation (21). In several models of lung injury after O_3_ exposure, free iron was identified as the cause of oxygen radical generation and lipid membrane peroxidation (31). Thoroughbreds racing under hazardously high O_3_ concentrations had decreased race performance (22), but an association between O_3_ exposure and markers of lung injury was not investigated. In mice, O_3_ exposure caused lung damage, alterations to surfactant protein activity (32) and neutrophilic inflammation in the lower airway (33). Experimentally, O_3_ inhalation in humans has been associated with lung inflammation and increased lower airway neutrophils (34). Increased ambient O_3_ exposure has also been associated with human mortality from general (35) and lung-related causes (36), particularly in association with increased ambient temperature (35). But, despite the evidence of ozone's deleterious effects on the lung, the association between O_3_ exposure and human asthma exacerbation (7) is considered tenuous (32, 37).

Decreased temperature has been associated with lower airway inflammation in horses (38–40). Increased ambient temperature, in conjunction with increased humidity and pollen concentration, was associated with worse disease exacerbation in horses with recurrent airway obstruction (41). In humans, a change in temperature was identified as having an independent effect on asthma exacerbation after controlling for the effects of ambient pollutants (42, 43). However, whether temperature has an independent effect on asthma exacerbation is unclear. Cold air exposure is associated with bronchoconstriction, particularly in association with exercise (44), but decreased ambient temperature was associated with asthma exacerbation only among poorly controlled asthmatics (45). Increased ambient temperature and asthma exacerbation in humans appeared to be mediated through the relationship between higher temperature and increased fine (46) and coarse particulate matter of a median aerodynamic diameter ≤ 10 μm [PM_10_; (47)]. Therefore, it has been recommended to assess for interaction between temperature and the concentration of various types of particulate matter before concluding that temperature has had an independent effect on asthma exacerbation (46).

In recognition of the relationship between ambient pollution and adverse health outcomes, the Ontario provincial government provides a free online tool, the Air Quality Health Index^1^ (AQHI). This tool is used by people with lung disease, including asthma, to decide how much outdoor activity is safe, particularly when air pollution is high^2^. The AQHI is a scale from 1 to 10^+^ where an AQHI value of one represents a low risk and 10^+^ indicates a high risk of exacerbation of lung disease secondary to ambient air pollution exposure^2^ (27). There are 39 Air Quality Ontario ambient air monitoring stations located around the province^3^ that measure and collect data on the concentrations of PM_2.5_ (μg/m^3^), NO_2_ (ppb), and O_3_ (ppb)^2^. These data are entered into a formula (27) from which a single, unitless, and geographically-specific AQHI value is generated.

Among asthmatic Ontarians, a one-point rise in the AQHI value predicted an increased risk of presenting to a health care provider for assessment of asthma exacerbation (25, 26). Children presented on the same day as the increase in AQHI, whereas adults presented with a lag of as many as 9 days after the AQHI increase (26). This pattern was attributed to children being more sensitive to the effects of air pollution and adults having a delay in the effects of the exposure (26).

There are relationships between an increase in AQHI and asthma exacerbation among people, and with fine particulate matter (20), O_3_ (21), temperature, and lower airway inflammation in horses (38, 39, 41). We wanted to determine whether similar relationships existed between these factors and a risk of finding lower airway inflammation in Ontario horses. Therefore, we assessed whether weekly mean ambient temperature, or an increase in the weekly mean value of PM_2.5_, NO_2_, and O_3_ was associated with the risk of finding increased proportions of one or a combination of neutrophils, eosinophils, and mast cells in BALF samples of Ontario horses. An additional goal was to detect whether a positive relationship could be identified between the AQHI and evidence of lower airway inflammation. If yes, then the AQHI could be used by horse industry personnel to modulate training schedules in order to mitigate a horse's risk of developing lower airway inflammation.

## Materials and Methods

### Data Collection and Handling

**Figure 1:**
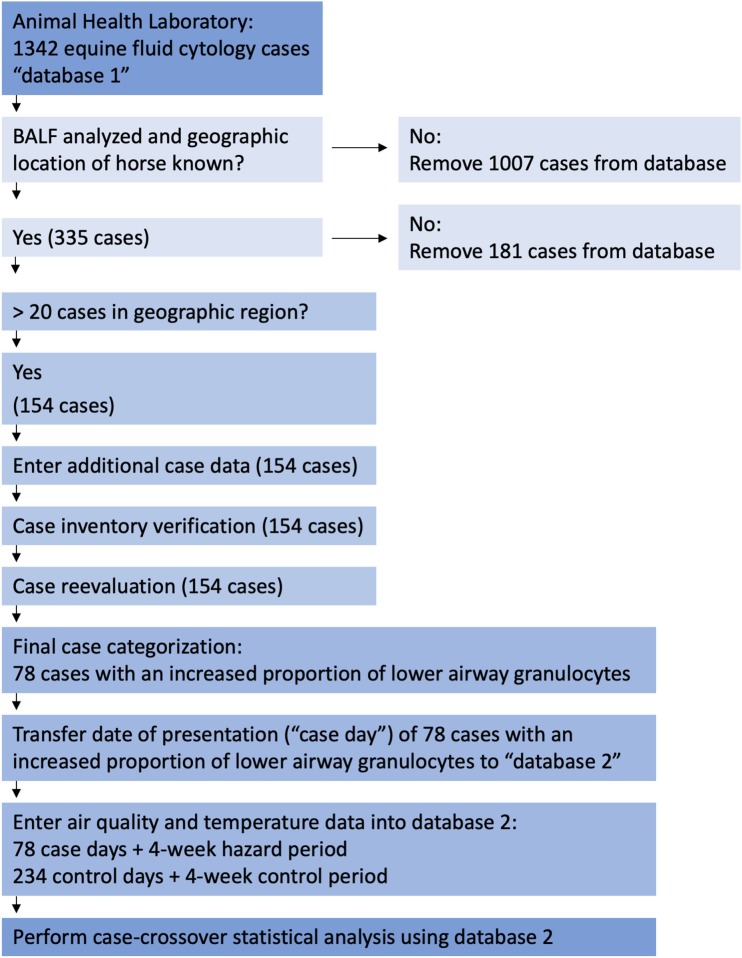
Flow chart of Animal Health Laboratory of Guelph database search, case selection methods, and data analysis. AHL, Animal Health Laboratory of Guelph; BALF, bronchoalveolar lavage fluid.

We searched the confidential database and microscopic slide archive of the Animal Health Laboratory, Guelph, Ontario for equine cases that included BALF analysis between May 8, 2007 and March 14, 2017 (Figure 1). This database originated in May of 2007 and is still in use. The search terms included“equine,” “fluid,” and “cytology” and the query yielded 1,342 cases, the majority of which originated from southern Ontario^4^ a geographic region of ~140,000 km^2^^5^. The initial data file, “database 1,” included case origin, that is, a private veterinary clinic or the Ontario Veterinary College, case submission number, date, species, breed, age, gender, animal name, owner or agent name, postal code, case history, and generally, but not always, the anatomic location from which the sample was obtained, for example, lung, trachea, joint, abdomen, etc. Cases were refined to include only BALF submissions using information found within database 1, or by using the case submission number to retrieve and assess case history and results. The same method was used to determine whether the case originated from a private clinic or the Ontario Veterinary College. This selection process yielded 335 BALF cases from locations scattered around the province of Ontario between June 13, 2007 to February 15, 2017. The geographic location of these 335 horses was determined by the trainer's stabling facility at the time the horse was presented, or if that was unknown, the postal code of the trainer or owner. For Standardbred racehorses, the trainer was confirmed by searching Standardbred Canada's database^6^ as the trainer is more likely to live closer to their training center than the owner. The next case selection process involved eliminating cases from geographic regions with fewer than 20 BALF submissions over the ten-year search period. Our rationale was that because we would use a case-crossover study design, meaningful results could not be generated from small concentrations of horses over so broad a period (48). This process resulted in 154 cases located within a 30-km radius^4^ of either Guelph or Kitchener, Ontario. As these two cities are 22 km apart^7^, the study area was ~42 km from east to west and 30 km from north to south^8^ Both cities have similar air quality conditions, and each contains a provincial air quality monitoring station^9^ and a federal government^10^ ambient temperature monitoring station.

Of the 154 cases identified in the Guelph-Kitchener region, 64 were submitted by a private veterinary clinic, and 90 by the Ontario Veterinary College. The next step in data handling (Figure 1) was to add additional case data to database 1 including history, bacterial culture results, present in 23 cases, original differential cell count, and original interpretation. In many cases a 400-cell differential count (49, 50) had not been performed during the original analysis, nor had hemosiderophages consistently been enumerated as part of the differential cell count. Therefore, a 400-cell differential count (49, 50) including hemosiderophage enumeration, was performed on a Wright's stained cytocentrifuge preparation or a sediment preparation if the former was not available. This permitted us to differentiate cases that had increased proportions of lower airway granulocytes and evidence of airway bleeding, from cases where hemosiderophages were identified in the absence of granulocytic inflammation. After repeating the differential cell counts, case information was evaluated and each case was assigned to one of the following categories: (1) insufficient sample for analysis or insufficient data to permit interpretation; (2) no cytologic abnormalities; (3) hemosiderophages in the absence of increased proportions of neutrophils, eosinophils, or mast cells; (4) pneumonia, lung abscess, or current history of fever; (5) current or previous diagnosis of recurrent airway obstruction or; (6) increased proportions of one or a combination of neutrophils, eosinophils, and mast cells, as indicators of lower airway inflammation. The definition of increased proportions of granulocytes followed that used for the cytologic portion of the diagnosis of IAD, namely, an increase in any one or a combination of neutrophils >5% (51) but <25%, eosinophils >1%, mast cells >2% (3) (Table 1).

**Table 1:** Classification of case data from 154 horses living within a 30-km radius of either Guelph or Kitchener, Ontario (2007–2017).

**Case classification based on history and BALF cytologic interpretation**	**Total no. of cases**	**Mean (range) NCC (×10^**9**^/L)**	**Mean, median (range) Neuts (%)**	**Mean, median (range) Eos (%)**	**Mean, median (range) Mast (%)**	**Mean, median (range) Hemosid (%)**
1. Insufficient sample for diagnosis	7	–	–	–	–	–
2. No cytologic abnormalities	7	0.41 (0.20–0.70)	1.5, 0.97 (0–4)	–	1, 1 (0–1)	–
3. Hemosiderophages No granulocytic inflammation	15	0.51 (0.38–1.6)	3, 3 (2–5)	0, 0 (0–1)	1, 1 (0–2)	9, 6 (2–36)
4. Pneumonia, lung abscess, fever	21	0.84 (0.28–5.9)	54, 55 (21–94)	1.3, 5.5 (0–14)	0.8, 4 (0–6)	Rarely noted
5. Recurrent airway obstruction	26	0.70 (0.28–1.2)	38, 35 (14–70)	0.1, 0 (0–2)	1.3, 0 (0–9)	–
6. Increased proportion of lower airway granulocytes	78	0.65 (0.23–1.6)	5, 3 (0–15)	2, 0 (0–31)	4, 3.8 (0–10)	7, 1.4 (0–47)

*Classification based on history and cytologic interpretation of bronchoalveolar lavage fluid. BALF, Bronchoalveolar lavage fluid; Eos, eosinophils; Hemosid, hemosiderophages; Mast, mast cells; Neuts, neutrophils; No., number; NCC, nucleated cell count*.

**Figure 2:**
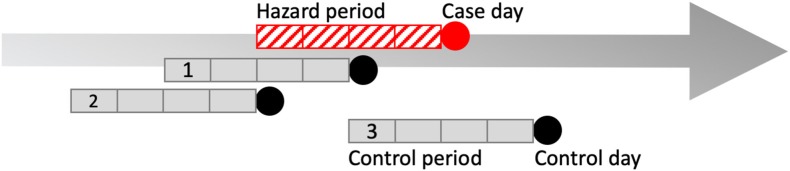
Pictorial representation of one possible case and control period arrangement in a case-crossover design. Four-week hazard period (red hatched box). Case day (red circle), and three 4-week control periods (gray boxes) and control days (black circles) compose the data from one case. The case-crossover design is a type of observational study where each individual serves as its own control, i.e., they are “self-matched” (44). The case-crossover study design is used to estimate the odds ratio of an individual experiencing an acute “event” immediately following exposure to a hazard over a defined period (44). Because controls are self-matched, it means the individual also has the potential for exposure to the hazard during the control periods (44). In our study, the exposures were ambient pollutants and temperature over a 4-week hazard period immediately prior to presentation. The day that bronchoalveolar lavage fluid was obtained was selected as the case day. To control for day-of-week effect in the model, control days consisted of all days falling on the same day of the week and year as the case day, within 4-weeks of case day occurrence. For example, if a horse presented on a Monday, control days were three different Mondays within a 4-week period of the case day during the same year. Therefore, matched sets for one finding of increased proportions of lower airway granulocytes consisted of one case and three control periods as shown above, or any other combination of pattern so long as all control periods fit within 4 weeks of case occurrence. Another method used to reduce bias in the statistical model was that control periods were randomly directed, meaning that control periods could either precede, follow, or straddle the hazard period. This may seem counterintuitive but if an exposure during the control period were meaningful, the horse could have had mildly increased proportions of lower airway granulocytes identified on a day other than the case day.

This process resulted in 78 cases that had an increased proportion of lower airway granulocytes. The presentation date of these 78 cases was transferred to a new database, “database 2.” Control dates, ambient pollutant, and temperature data were added to database 2 following the steps outlined in Sections Air Quality Health Index—Component Ambient Pollutants and Formula and Data Analyses below, and in Figures 1, 2. Raw data is available from the corresponding author on request.

#### Air Quality Health Index—Component Ambient Pollutants and Formula

Mean daily values of PM_2.5_, NO_2_, and O_3_ were obtained from the Air Quality Ontario ambient air monitoring stations located in Guelph and Kitchener^11^. Mean AQHI was calculated using mean daily values of PM_2.5_, NO_2_, and O_3_ according to the formula (27):

$$\begin{array}{l} AQHI=10÷10.4\times (100\times (e^{\left(0.000871\times {\text{NO}}_{2}\right)}+e^{\left(0.000537\times {\text{O}}_{3}\right)}\\ +e^{\left(0.000487\times \text{PM}2.5\right)}-3)) \end{array}$$

Mean daily ambient temperature values were obtained from the Environment Canada weather stations located in Guelph and Kitchener^12^. AQHI and temperature data were collected from May 13, 2007 to March 15, 2017. Because we based our definition of increased proportions of one or a combination of neutrophils, eosinophils, and mast cells on the cell percentages used in the diagnosis of IAD (3, 51), and because the clinical diagnosis of IAD requires a chronicity of 4 weeks (3), we collected air pollution and temperature data for lag periods of 1–4 weeks prior to the horse's presentation. We considered this 4-week period the hazard period, as explained in Case-Crossover Study Design below.

#### Data Analyses

##### Case-crossover study design

The case-crossover study design compares exposures identified during a defined hazard period to the case period, and is used to evaluate the relationship between acute, short-term exposures, and statistically rare outcomes (52). The hazard period occurs immediately prior to the case period, and self-matched control periods are defined for each individual case period (Figure 2). Exposures that increase the probability of case occurrence are expected to occur with greater frequency during the hazard period (52).

In our study, the case period, or “case day,” was the day that the horse presented for respiratory tract examination including BALF analysis through which increases in the proportions of one or a combination of neutrophils, eosinophils, and mast cells was identified. The hazard period was defined as the 4 weeks leading up to the date of the case day. The hazard period included 1–4 week lagged time periods and each horse's case day was self-matched to 3 control days. Control days consisted of all days falling on the same day of the week and in the same year as the case day within 4 weeks of case occurrence, and the control period consisted of the 4-week time period leading up to each control day (Figure 2). Therefore, this was a time-lagged matched design in which three control periods were created and matched by day of week to each case day. This approach controls for day of week, seasonality, exposure time trends and inter-subject variability, and is a method used to reduce bias in conditional logistic regression estimates (52, 53). Bias was further reduced by using random directionality for control period selection, such that control periods could follow, precede, or straddle the hazard period [Figure 2; (53)]. Consider the example of a case that occurred on November 2nd with randomly directed control days occurring on October 19th, October 26th, and November 9th. Each of these days has 1–4 week lagged exposures associated with them. The purpose of this portion of the analysis is to determine whether the exposure levels, including lagged exposures, are different for the case day compared with the exposure levels, including lagged exposures, for the control days.

Predictor variables, including 1–4 week lagged exposures were assessed for associations with the finding of increased proportions of granulocytes on BALF cytologic examination. Odds ratios for the cytologic finding of increased granulocytes were estimated using univariable and multivariable conditional logistic regression models, and a 95% CI was calculated (**Tables 3**, **4**). The multivariable model was built using a backward elimination selection process. With the exception of AQHI, all exposure variables with a *p* < 0.2 were included in the initial model. Criteria for inclusion in the final model included a *p* < 0.05 as well as the model fit statistics, Akaike information criterion and Bayesian information criterion. These statistics are used to determine whether a model is a better fit with the inclusion, rather than the exclusion, of the variable. The data were also scrutinized for the presence of confounding variables. This analysis was conducted by examining the remaining variables for any significant changes both including and excluding the potentially confounding variable from the statistical analysis. Interaction terms were created and assessed for inclusion in the model. Statistical analyses were conducted using Stata 14 software (StataCorp. 2015. *Stata Statistical Software: Release 14*. College Station, TX: StataCorp LP).

##### Proportional morbidity calculation

There was no data on the total horse population of Ontario over the 10 years of this study, therefore, the prevalence of the finding of increased proportions of lower airway granulocytes could not be calculated. Instead, we calculated the proportional morbidity, a disease rate calculation that is used when the true population number is not known (54). Proportional morbidity is calculated by dividing the number of disease-of-interest cases by the number of all cases examined. In our study the “disease” was defined as the finding of increased proportions of granulocytes on BALF cytologic examination.

## Results

Within the study area, the total number of horses that presented to a veterinarian for examination of the respiratory tract, which included BALF cytologic examination, was 154 (Table 1). The number of horses that had increased proportions of lower airway granulocytes was 78 (Table 2). Therefore, between June 13, 2007 and February 15, 2017, the proportional morbidity of increased lower airway granulocytes in the area of Guelph and Kitchener was 51%. Of the 78 horses that had increased proportions of lower airway granulocytes, 58 were racing or training Standardbreds. The average age of Standardbreds was 3.75 years. The remaining 20 horses were a mixture of Warmbloods (*n* = 5), Thoroughbreds (*n* = 3), retired Standardbreds used as teaching horses at the Ontario Veterinary College (*n* = 3), and a mixture of other horse breeds (*n* = 9). The average age of the cohort of 20 horses was 10.6 years. The nucleated cell count of BALF was similar across cytologic categories (Table 1). Of the 78 horses with increased lower airway granulocytes, mastocytic inflammation was the most common form identified (Table 2). Three Standardbred racehorses with an average age of 2.9 years had mixed eosinophilic and mastocytic inflammation (Table 2). These three horses had eosinophil proportions of 20−30%. Hemosiderophages were variably prominent by light microscopy, but no iron stains were applied to facilitate their identification. The average proportion of hemosiderophages in horses that had increased lower airway granulocytes was 7%, and in horses with hemosiderophages in the absence of granulocytic inflammation was 9% (Table 1).

**Table 2:** Classification of inflammatory cell types from 78 horses with an increased proportion of lower airway granulocytes.

**Classification based on results of BALF 400-cell differential count**	**Total no. of cases**	**Mean (Range) NCC (×10^**9**^/L)**	**Mean, Median (Range) Neuts (%)**	**Mean, Median (Range) Eos (%)**	**Mean, Median (Range) Mast (%)**	**Mean, Median (Range) Hemosid (%)**
1. Neutrophilic	13	0.62 (0.35–1.3)	8, 9 (6–13)	0, 0 (0–1)	1, 1 (0–2)	2, 1 (0–20)
2. Neutrophilic + mastocytic	12	0.73 (0.3–1.3)	10, 11 (6–15)	0, 0 (0–1)	5, 4 (3–10)	12, 4 (0–47)
3. Neutrophilic + eosinophilic	1	0.64	11	2	0	44
4. Mastocytic	35	0.65 (0.14–1.6)	2, 2 (0–5)	0, 0 (0–1)	5, 4 (3–10)	6, 2 (0–31)
5. Mastocytic + eosinophilic	14	0.63(0.3–1.1)	2, 2 (1–4)	11, 9 (2–31)	6, 7 (3–9)	9, 8 (0–31)
6. Eosinophilic	3	0.93 (0.7–1.3)	2 (1)	4 (2–6)	2(2)	1 (0–1)

*Horses lived within a 30-km radius of either Guelph or Kitchener, Ontario (2007–2017). BALF, Bronchoalveolar lavage fluid; Eos, eosinophils; Hemosid, hemosiderophages; Mast, mast cells; Neuts, neutrophils; No., number; and NCC, nucleated cell count*.

The case-crossover analysis of 78 cases with an increased proportion of lower airway granulocytes (Tables 3, 4) demonstrated statistically significant associations with lagged weekly mean PM_2.5_ and NO_2_ but not with the AQHI value or O_3_. No confounding variables or interactions were identified in any of the analyses. There was an 11% increased risk of identifying higher proportions of lower airway granulocytes 2 weeks after a 1 μg/m^3^ rise in weekly mean PM_2.5_ (*p* = 0.04, 95% CI = 1.01–1.22) in the multivariable analysis (Table 4). Similar results were found in the univariable analysis (Table 3). The univariable and multivariable analyses both identified a higher risk of finding increased proportions of lower airway granulocytes 3 weeks after a 1 ppm increase in weekly mean NO_2_ (Tables 3, 4). However, with a 4-week lag, the univariable analysis also revealed that for each unit increase in weekly mean NO_2_ there was a reduced risk of identifying increased airway granulocytes on BALF cytologic examination (Table 3).

**Table 3:** Univariable associations between weekly average ambient exposures and increased proportions of lower airway granulocytes (*n* = 78, 2007–2017).

**Exposure**	**Odds ratio**	**95% CI**	***p*-value**
**Mean ambient temperature (°C)**			
0-week lagged	1.01	0.92–1.12	0.82
1-week lagged	1.10	0.99–1.21	0.07
2-week lagged	1.09	0.98–1.20	0.10
3-week lagged	0.96	0.88–1.04	0.33
4-week lagged	0.95	0.86–1.04	0.28
**Mean NO**_**2**_			
0-week lagged	0.92	0.80–1.06	0.24
1-week lagged	0.89	0.77–1.03	0.13
2-week lagged	1.04	0.91–1.18	0.59
3-week lagged	**1.18**	**1.03–1.35**	**0.02**
4-week lagged	**0.82**	**0.69–0.97**	**0.02**
**Mean O**_**3**_			
0-week lagged	1.03	0.96–1.10	0.47
1-week lagged	1.00	0.93–1.08	0.95
2-week lagged	1.04	0.97–1.11	0.26
3-week lagged	0.99	0.93–1.05	0.72
4-week lagged	1.00	0.94–1.07	0.94
**Mean PM**_**2.5**_			
0-week lagged	0.99	0.89–1.10	0.85
1-week lagged	0.94	0.84–1.05	0.26
2-week lagged	**1.11**	**1.01**–**1.22**	**0.03**
3-week lagged	1.02	0.93–1.12	0.64
4-week lagged	1.01	0.92–1.12	0.78
**Mean AQHI**			
0-week lagged	0.90	0.40–2.01	0.80
1-week lagged	0.59	0.25–1.38	0.23
2-week lagged	1.93	0.92–4.04	0.08
3-week lagged	1.49	0.72–3.08	0.28
4-week lagged	0.72	0.34–1.53	0.39

*Statistically significant associations (p <0.05) are in bold-face type. Zero- to four-week lagged mean values for ambient temperature, nitrogen dioxide (NO_2_), ozone (O_3_), fine particulate matter <2.5 microns diameter (PM_2.5_), and Air Quality Health Index (AQHI). Odds ratio, 95% confidence interval (CI), and p-value of the estimate*.

**Table 4:** Final multivariable model results showing the associations between weekly average ambient exposures and increased proportions of lower airway granulocytes (*n* = 78, 2007–2017).

**Exposure**	**Odds Ratio**	**95% CI**	***p*-value**
**Mean ambient temperature (°C)**			
1-week lagged	**1.13**	**1.02–1.26**	**0.02**
**Mean NO**_**2**_			
3-week lagged	**1.24**	**1.08–1.43**	**0.03**
**Mean PM**_**2.5**_			
2-week lagged	**1.11**	**1.01–1.22**	**0.04**

*Statistically significant associations (p <0.05) are in bold-face type. Mean values for ambient temperature, nitrogen dioxide (NO_2_), fine particulate matter <2.5 microns diameter (PM_2.5_). Odds ratio, 95% confidence interval (CI), and p-value of the estimate*.

In the multivariable model, with a 1-week lag, a single degree Celsius increase in weekly mean ambient temperature was associated with a 13% increase in the risk of identifying an increase in the proportion of lower airway granulocytes on BALF cytologic examination (*p* = 0.02, 95% CI = 1.02–1.26, Table 4).

## Discussion

This is the first investigation of the relationship between ambient risk factors and the cytologic identification of increased proportions of lower airway granulocytes in horses.

Increased mast cells accounted for the majority of cases with increased lower airway granulocytes. Increased mast cells were positively associated with β-glucan exposure (5), but β-glucan was not measured in our study. Furthermore, we cannot speculate whether there was an association between mast cells and a particular ambient pollutant because the number of cases was too small to assess by the case-crossover study design.

We identified a small number of young Standardbreds in training with mixed mastocytic and eosinophilic inflammation that had greater increases in airway eosinophils than the majority of horses with eosinophilic lower airway inflammation alone. On a concurrent complete blood count, one of these horses had absolute eosinophil counts within the reference interval. The other two horses did not have a complete blood count performed, and none had a fecal examination performed. Previous investigators also identified a small cohort of young Standardbreds with considerably higher eosinophil proportions than the majority in their study (9, 11). Mast cells were either not discussed (9) or not discussed in association with the finding of increased eosinophils (8, 11) in these studies. However, similar to these investigators (9, 11), we considered allergic airway inflammation a more likely cause of the relatively higher airway eosinophils than lung worm infection (8).

In our study, exposure to higher PM_2.5_ and NO_2_ was associated with a greater risk of identifying increased proportions of lower airway granulocytes with a 2- and 3-week exposure lag, respectively (Table 4). Previous work showed that exposure to PM_2.5_ was associated with increased tracheal mucus and tracheal neutrophils in horses (20), as well as neutrophilic lung inflammation in mice (55). Increased ambient PM_2.5_ was identified as a risk factor for asthma exacerbation, particularly among children (56) in both warm and cold seasons (30). The effects of NO_2_ on horse lung health have not been investigated, but NO_2_ was implicated as increasing allergic asthma susceptibility in rat and mouse models after prolonged exposure (57, 58), and NO_2_ is considered a major contributor to asthma exacerbation in children in both developed (56) and developing nations (23).

The WHO guideline for daily mean PM_2.5_ exposure is a maximum of 25 μg/m^3^ (28). For NO_2_, the maximum hourly exposure guideline is 200 μg/m^3^ (28). In our study region, no 2-week lagged weekly mean PM_2.5_ value was >25 μg/m^3^; indeed, the majority of weekly means were <10 μg/m^3^. When converted from ppb^13^, the 3-week lagged weekly mean NO_2_ was most commonly <13 μg/m^3^ and none exceeded 38 μg/m^3^. However, we observed statistically significant relationships between increased weekly mean PM_2.5_ and NO_2_ and an increased proportion of lower airway granulocytes on BALF cytologic examination (Tables 3, 4). One explanation is that a synergistic effect exists between PM_2.5_, NO_2_, and other types of particulates, such as PM_10_ (59, 60), dust (5, 38, 61), or pollen (41) that were not accounted for in our model. Alternately, it could be that similar to their effects on humans, PM_2.5_ (46) and NO_2_ (62) have deleterious effects on the equine lung at lower concentrations than are suggested by the WHO guidelines (28).

In the univariable analysis, each unit increase in 4-week lagged weekly mean NO_2_ was associated with a reduced risk of identifying an increased proportion of lower airway granulocytes. There was an increased risk of finding higher proportions of lower airway granulocytes 3 weeks after a rise in weekly mean NO_2_ (Table 3). These results may imply that the peak effect of NO_2_ occurs between weeks 3 and 4, as the positive relationship between 2-week lagged mean NO_2_ and the finding of higher proportions of lower airway granulocytes remained in the multivariable analysis (Table 4).

An increase in weekly mean ambient temperature was associated with a 13% increase in the risk of identifying higher proportions of lower airway granulocytes on BALF cytologic examination 1 week after the temperature increase occurred (Table 4). The WHO guidelines do not discuss the effects of temperature on health (28). An interaction between increased temperature and PM_2.5_ in association with human asthma exacerbation was observed (46), however, we did not identify a similar relationship in our data analysis. An association between increased ambient temperature and pollen concentration was implicated the exacerbation of recurrent airway obstruction in horses (41) and between temperature and increased PM_10_ in the exacerbation of human asthma (47). Measurements of these classes of particulates were not available to us, so we could not examine their relationship to temperature, but future studies should attempt to investigate these types of interactions to thoroughly examine the role of temperature in exacerbation of lower airway inflammation in horses.

Ontario is humid in the summer, but humidity was not included our model because the weather database was incomplete. However, this would be an important variable to include in future models, because increased humidity was believed to be associated with increased temperature and severity of clinical signs in horses with recurrent airway obstruction (41).

Although our statistical model controlled for seasonality, 63% of cases were presented during the warm season of April to September (27). Therefore, our data contained insufficient cool-season case numbers to meaningfully assess the effects of colder temperatures on the finding of increased proportions of lower airway granulocytes. As 74% of horses with increased lower airway granulocytes were Standardbred racehorses, summer presentation may have related to poor performance noted during training in preparation for the summer stakes racing season. Mild lower airway inflammation is most commonly reported in younger horses (3), and the majority of animals assessed in this study were <4 years of age. Therefore, morbidity may have also reflected age-related factors such as mixing with new horses and increased susceptibility to viral infection (1, 63).

The WHO suggests an independent effect of O_3_ on human health and offers an 8 h exposure guideline of 100 μg/m^3^, or 51 ppb^13^ (28). Whether O_3_ has an effect on asthma development or exacerbation is undefined (32, 37). Stall-housed horses exposed to 0.5 ppm O_3_ for 12 h, had increased free iron, and markers of oxidant injury and inflammation in BALF (21). Rats exposed to 0.8 ppm O_3_ for 12 h had alterations to surfactant proteins (32). Mice exposed to 0.7 ppm O_3_ for 72 h (33) and humans that inhaled 0.3 ppm O_3_ for 2 h with intermittent exercise (34) had neutrophilic lower airway inflammation. In our study, mean daily O_3_ ranged from 7.43–57.92 ppb. While less than the WHO guideline number, O_3_ concentrations in our study were higher than experimental O_3_ exposure values (21, 32, 33). However, we did not identify an association between higher ambient O_3_ and increased proportions of lower airway granulocytes. In Ontario, O_3_ values tend to be higher between May and September and between midday and early evening^14^. Although most of our horses presented during the warm months, most Standardbreds are exercised before noon and race from early to late evening. So, it could be that horses in our study avoided exercising during the worst exposure period when the deleterious effects of exposure would presumably be most pronounced.

Increased AQHI values have been associated with asthma exacerbation in children and adults with variable lag times after the increase (25, 26). In our study, an increase in 2-week lagged mean AQHI approached statistical significance at *p* = 0.08, however, the 95% confidence interval of 0.92–4.04 was broad. This suggests that case numbers were insufficient to adequately probe the relationship as some of the individual pollutants used in calculating the AQHI value had a statistically significant association with the finding of increased lower airway granulocytes, albeit during different weeks (Tables 3, 4).

We did not know whether the 78 horses with increased lower airway granulocytes also received a clinical diagnosis of IAD. However, they were presented for examination of the respiratory tract including bronchoalveolar lavage and did have increased lower airway inflammatory cells similar to those expected in horses with IAD. More complete data including onset of clinical signs and clinical diagnosis could have strengthened the work by linking ambient pollution directly to a diagnosis of IAD. Alternately, the examination of a surrogate measure such as decreased race performance could have been included, however, this action would have reduced the number of cases for analysis. A prospective study could provide control over these critical pieces of data and could allow examination of potentially confounding variables such as treatment. However, a prospective study would require a comprehensive case recruitment and maintenance strategy to ensure adequate case numbers.

At 51%, the proportional morbidity of identifying increased lower airway granulocytes indicates that lower airway inflammation is an important target for prospective studies of its association with ambient exposures. To more clearly define the relationship of temperature to lower airway inflammation in horses, it would be important to examine the data for interactions between temperature and particulates including PM_10_ and pollen, and not just PM_2.5_. The utility of the AQHI in relation to lower airway inflammation in horses was not fully explored in this study because of insufficient case numbers. However, our results extend the present understanding of the health effects of PM_2.5_ and NO_2_ exposure in horses by providing evidence that ambient pollutants are associated with lower airway inflammation, particularly in Standardbred racehorses in the Guelph-Kitchener region of Ontario.

## Data Availability Statement

Datasets are available on request. Data supporting the conclusions of this article have been cleared of any patient-identifying information and can be made available by JB-M, without undue reservation, to any qualified researcher.

## Ethics Statement

Ethical review and approval were not required as no testing of animals was conducted for the purposes of this study and all case material was obtained during a regular medical examination. Written informed consent for participation was not obtained from horse owners or agents because, as a retrospective study, obtaining informed consent was not possible.

## Author Contributions

GB database curation: final Air Quality Health Index and temperature data entry, performed all statistical analyses. AG methodology, database formatting, and analysis. QM initial database curation: entered initial Air Quality Health Index data and case data. BL and KM entered additional Air Quality Health Index and temperature data. BL restructured database. DH performed initial statistical analyses. JH assisted in determining horse location. JB-M conceptualized all research, acquired funding, directed project work, and performed all 400-cell differential counts. All authors contributed to preparation of the manuscript.

## Conflict of Interest

The authors declare that the research was conducted in the absence of any commercial or financial relationships that could be construed as a potential conflict of interest.

## Footnotes

^1^http://www.airqualityontario.com/aqhi

^2^http://www.airqualityontario.com/science/aqhi_description.php

^3^http://www.airqualityontario.com/aqhi/locations.php

^4^https://www.feddevontario.gc.ca/eic/site/723.nsf/eng/01970.html?OpenDocument

^5^https://www.ontariotravel.net/en/plan/tools-and-tips/facts-about-ontario

^6^https://trackit.standardbredcanada.ca/

^7^https://www.distance.to/Kitchener/Guelph,Ontario,CAN

^8^https://www.mapdevelopers.com/draw-circle-tool.php

^9^http://www.airqualityontario.com/aqhi/locations.php (accessed May 3, 2019).

^10^http://climate.weather.gc.ca (accessed May 3, 2019).

^11^http://www.airqualityontario.com/history/index.php (accessed May 3, 2019).

^12^http://climate.weather.gc.ca (accessed May 3, 2019).

^13^http://www.apis.ac.uk/unit-conversion

^14^http://www.airqualityontario.com/science/pollutants/ozone.php

## Abbreviations

BALF: Bronchoalveolar lavage fluid
PM_10_: Coarse particulate matter ≤ 10 microns diameter
CI: Confidence interval
PM_2.5_: Fine particulate matter ≤ 2.5 microns diameter
IAD: Inflammatory airway disease
NO_2_: Nitrogen dioxide
O_3_: Ozone
WHO: World Health Organization.
